# Intraperitoneal administertion: compatibility and stability of common antibiotics in amino acid-based peritoneal dialysate

**DOI:** 10.3389/fphar.2026.1808066

**Published:** 2026-07-02

**Authors:** Bohua Zhang, Lichao Zhong, Xueli Zhou, Hui Zhong, Yuqin Xiong, Hongliu Yang, Ruoxi Liao

**Affiliations:** Department of Nephrology, West China Hospital of Sichuan University, Chengdu, Sichuan, China

**Keywords:** amino acid, antibiotics, compatibility, dialysate, peritoneal dialysis

## Abstract

**Background:**

The preferred method of antibiotic administration for peritoneal dialysis-related peritonitis is intraperitoneal. However, there are few studies on the compatibility of antibiotics in amino acid-based peritoneal dialysate.

**Methods:**

Our study employed High Performance Liquid Chromatography (HPLC) and amino acid analyzer to evaluate the stability of different antibiotics in amino acid-based peritoneal dialysate. We also used the two-dose method to detect the antimicrobial potency of gentamicin.

**Results:**

Gentamicin, ciprofloxacin, heparin Sodium and fluconazole showed excellent stability under different conditions (14 days at 4 °C: ≤3.57%, 14 days at 25 °C: ≤3.77%). β-Lactam antibiotics including meropenem, ceftazidime, cefotaxime, and aztreonam showed significant degradation, especially at high temperature (37 °C)(14 days at 4 °C: ≤59.98%, 14 days at 25 °C: 19.06%–84.97%, except for aztreonam demonstrating degradation at 48 h at 37 °C (18.51%–41.88%). Among them, meropenem was the least stable (14 days at 4 °C: 59.98%, 14 days at 25 °C: 84.97%). Low concentration vancomycin is relatively stable, while medium and high concentration vancomycin has poor stability (14 days at 4 °C 500 mg/L: 45.8%, 1 g/L 27.7%). Most amino acids remained basically stable under different antibiotic environments. We counted the degradation rate of all amino acids under different conditions. The maximum degradation rate appeared on glycine, but it was only 8.21%. Finally, we also found that the antibacterial efficacy of gentamicin in acid-based peritoneal dialysate was enhanced.

**Conclusion:**

Most antibiotics are stable in amino acid-based peritoneal dialysate. However, β-lactams (especially meropenem, ceftazidime, and cefotaxime) and medium-to-high dose vancomycin showed significant degradation and require prompt administration. The nutritional amino acid profile in dialysate remains largely unaffected. Gentamicin demonstrates enhanced antibacterial activity in the amino acid-based peritoneal dialysate.

## 1 Introduction

Peritoneal dialysis is an important renal replacement therapy. Peritonitis is a common complication of peritoneal dialysis (Bartolomeo et al., 2022). ISPD guidelines recommend intraperitoneal antibiotic administration as the first choice for patients with peritonitis (Li et al., 2016). Antibiotics can be added to glucose and icodextrin peritoneal dialysate, and their compatibility has been confirmed in previous studies (So et al., 2022). Amino acid-based peritoneal dialysate is a kind of dialysate which can be used to improve the nutritional status of patients. As a neutral peritoneal dialysate, it also helps to protect the peritoneum (Li et al., 2003; Chaudhary and Khanna, 2010). However, there is few relevant studies on the compatibility of amino acid-based peritoneal dialysate and antibiotics. When different antibiotics are mixed with amino acid-based peritoneal dialysate, drug and amino acids may be also different. Therefore, our study was aimed to explore the compatibility of amino acid-based peritoneal dialysate and antibiotics, and the stability of the two at different temperatures and storage times.

## 2 Methods

### 2.1 pH, osmotic pressure and appearance observation

We mixed amino acid-based peritoneal dialysate with different antibiotics, then measured the pH and osmotic pressure of dialysate at different temperatures and storage times. We also observed the appearance of dialysate including its colour and whether it is clear in different conditions.

### 2.2 Concentration detection

This study employed High Performance Liquid Chromatography (HPLC) and amino acid analyzer to evaluate the compatibility stability of amino acid-based peritoneal dialysate (Peritoneal Dialysis Solution, Lactate-G1.5% (Chengdu Qingshan Likang Pharmaceutical Co., Ltd., Chengdu, China)) with antibiotics. Each concentration measurement under a given condition was performed once, as this was a screening-level physicochemical stability study. The specific methods are as follows.

#### 2.2.1 Concentration detection of antibiotics and tryptophan/tyrosine

A C18 column (250 cm × 4.6 cm, 5 μm) was used with mobile phase A (0.02 mol/L sodium dihydrogen phosphate) and mobile phase B (acetonitrile). Gradient elution programs are listed in Supplementary Tables S1, 2. The detection wavelength was selected based on the target compound (e.g., 270 nm for tryptophan and tyrosine, 254 nm for ceftazidime). The injection volume was 10 μL (100 μL for amoxicillin and fluconazole). Test samples were directly injected, and reference solutions were prepared by dissolving antibiotic standards. Concentrations were calculated using the internal standard method (phenylalanine as the internal standard).

#### 2.2.2 Detection of other amino acids

A sulfonic acid-based strong cation-exchange sodium system column (4.6 mm × 150 mm, 7 μm) was employed with mobile phase A (citrate buffer, pH 3.45) and mobile phase B (citrate buffer, pH 10.85). The regeneration solution was sodium hydroxide, and the derivatizing agent was ninhydrin. The detection wavelengths were 570 nm and 440 nm, with an injection volume of 50 μL. The flow rate of buffer pump was 0.45 mL/min and the flow rate of derivative pump was 0.25 mL/min. Ninhydrin was selected as the derivatizing agent because it is the standard reagent for post-column derivatization in ion-exchange chromatography, offering excellent stability, high reproducibility, and compatibility with the detection wavelengths (570 nm for primary amino acids and 440 nm for proline). Gradient elution and temperature control programs are detailed in Supplementary Tables S3, 4. Test samples were diluted with 0.02 mol/L hydrochloric acid, and reference solutions were prepared by diluting amino acid peritoneal dialysis fluid. Concentrations were determined using the external standard method.

### 2.3 Gentamicin antimicrobial efficacy detection method

Although multiple antibiotics were evaluated for chemical stability, only gentamicin was selected for antimicrobial efficacy testing in our study for the following reasons: 1. Gentamicin is an empirical anti-infective drug recommended by ISPD guidelines; 2. Our study suggested that gentamicin and amino acid-based peritoneal dialysate are stable under all conditions.; 3. Previous studies have suggested that gentamicin combined with amino acids can enhance its antibacterial effect. Resource limitations prioritized the most clinically relevant and mechanistically interesting candidate. The antimicrobial potency of gentamicin was determined using the two-dose method, which compares the inhibition zone diameters of standard and test samples at two concentrations (high and low) to calculate relative bioactivity. We also performed three independent replicates for each sample under each condition, which applies to the efficacy assay only.

#### 2.3.1 Standard curve preparation

Gentamicin standard (1 mg/mL) was diluted to a series of concentrations (0.1, 1, 10, 100 μg/mL). Bacterial suspensions of *Staphylococcus aureus* (single bacteriostasis) (1 × 105 CFU/mL) were evenly spread on nutrient agar plates. After solidification for 5 min, 6 mm wells were punched, and 50 μL of each standard solution was added. Plates were incubated at 37 °C for 24 h, and inhibition zone diameters (mm) were measured. The standard curve was plotted with log C (logarithm of concentration) as the x-axis and inhibition zone diameter (D) as the y-axis, fitted to the equation:

D=a+b ·⁡log⁡C

#### 2.3.2 Sample assay

Test samples were filtered (0.22 μm) and diluted to high and low doses (e.g., 10 μg/mL and 100 μg/mL, matching the standard dose ratio). Inhibition zones were measured as above.

#### 2.3.3 Potency calculation

The potency (P) was calculated using the two-dose formula:

$$P=\text{antilog}\left[\left(\text{TH}+\text{TL}\right)-\left(\text{SH}+\text{SL}\right)/\left(\text{TH}-\text{TL}\right)+\left(\text{SH}-\text{SL}\right)\right]\times \log K$$

where TH and TL are inhibition zone diameters of the test sample at high/low doses, SH and SL are standard values, and K is the dose ratio (e.g., 10:1).

### 2.4 Statistical method

We measured the concentrations of various antibiotics and amino acids included at three different temperatures and four different times by the above method. We then imported the concentration data into an excel table, and used the formula ((initial concentration-detection concentration)/ initial concentration) to calculate the degradation rate in different environments. Lower degradation rate means better stability. We comprehensively evaluated the stability of antibiotics and amino acids by using the degradation rates in different environments.

## 3 Results

### 3.1 Appearance, pH, and osmolarity of Peritoneal Dialysis Solution

We found that the pH and osmolarity of all antibiotics were most stable at 4 °C, and slightly changed with time at 25 °C and 37 °C (pH ≤ ±0.15, osmolarity ≤±9 mOsmol/kg). For the appearance of peritoneal dialysate, we found that they remained stable for 14 days at 4 °C, while some samples showed color changes or precipitation at 25 °C after 14 days. A small amount of precipitation was observed in amoxicillin, fluconazole and aztreonam. A small amount of flocculent clindamycin, ceftriaxone, and agglomerates was observed in the combination of piperacillin and tazobactam. From the perspective of time, all samples showed stable pH, osmolarity, and appearance in the short term (within 48 h). Long-term storage at low temperature (4 °C) was more favorable for stability, while higher temperatures (25 °C and 37 °C) could lead to changes in appearance. The detailed results of pH, osmolarity, and appearance observations are summarized in Supplementary Table S5.

### 3.2 Drug concentration changes

#### 3.2.1 General trends

The concentration of all antibiotics was most stable at 4 °C and drug degradation was significantly faster at 37 °C compared to the other two temperatures. Most drugs showed a notable decline in concentration over time, especially after 14 days. For specific types of drugs, gentamicin, ciprofloxacin, heparin Sodium and fluconazole showed excellent stability under different conditions (14 days at 4 °C: ≤3.57%, 14 days at 25 °C: ≤3.77%). β-Lactam antibiotics including meropenem, ceftazidime, cefotaxime, and aztreonam showed significant degradation, especially at high temperature (37 °C)(14 days at 4 °C: ≤59.98%, 14 days at 25 °C: 19.06%–84.97%, except for aztreonam demonstrating degradation at 48 h at 37 °C (18.51%–41.88%). Among them, meropenem was the least stable (14 days at 4 °C: 59.98%, 14 days at 25 °C: 84.97%). Stability classification of antibiotics included in amino acid-based peritoneal dialysate can be seen in Table 1. The detailed degradation rate data of each antibiotic under different conditions can be seen in Supplementary Table S6. We can also see from the large types of drugs in the Table1, the stability of β-lactam antibiotics in amino acid dialysis solution seems to be poor.

**TABLE 1:** Stability classification of commonly used antibiotics in amino acid-based peritoneal dialysate.

Drug class	Drug name	Concentration range	Degradation rate at 25 °C (14 d)	Stability level	Recommendation
Aminoglycosides	Gentamicin	1000 U/mL	≤3.77%	Highly stable	Suitable for IP use, even with delayed administration
Quinolones	Ciprofloxacin	50 mg/L	0%	Highly stable	Suitable for IP use
Anticoagulants	Heparin sodium	500 mg/L	≤1.35%	Highly stable	Suitable for IP use
Triazole antifungals	Fluconazole	75–100 mg/L	≤0.1%	Highly stable	Suitable for IP use
Cephalosporins	Cefazolin	125–500 mg/L	14.9%–18.3%	Low stability	Use promptly after mixing
​	Ceftazidime	125–500 mg/L	28.4%–30.8%	Unstable	Use immediately
​	Cefotaxime	250–500 mg/L	28.9%–29.8%	Unstable	Use immediately
​	Cefepime	125–500 mg/L	11.7%–12.4%	Low stability	Use promptly
​	Ceftriaxone	500 mg/L	18.1%	Low stability	Use promptly
Penicillins	Amoxicillin	150 mg/L	– (Precipitation)	Unstable	Avoid IP use?
​	Ampicillin	125 mg/L	15.2%	Low stability	Use promptly
​	Piperacillin/tazobactam	1–4 g/L	2.5%–3.7%	Moderately stable	Use within short time
Carbapenems	Meropenem	125 mg/L	85.0%	Unstable	Not recommended for IP use
Monobactams	Aztreonam	250–500 mg/L	19.0%–23.6%	Unstable	Use immediately
Glycopeptides	Vancomycin	25 mg/L	41.2%	Unstable (low conc)	Use promptly
​	​	500 mg/L	51.5%	Unstable	Use immediately
​	​	1 g/L	44.5%	Unstable	Stable only at 4 °C
Lincosamides	Clindamycin	300 mg/L	– (no data at 14 d)	–	Not determined

Full degradation data at 4 °C, 25 °C, 37 °C over different time intervals as well as concentration-dependent data are provided in Supplementary Table S6. The definition of grading and temperature sensitivity is included in the Appendix.

#### 3.2.2 Concentration impact

The stability of most antibiotics is not affected by their concentration. Low concentrations of cefoperazone, cefazolin and aztreonam were slightly more stable over time. Low concentration vancomycin is also more stable. Medium concentration (500 mg/mL) showed the poorest stability (14 days at 25 °C 500 mg/L: 51.5%), followed by high concentration (14 days at 25 °C 1 g/L 44.5%). However, high concentration demonstrated special stability at C (27.7%). The detailed degradation rate data of different concentrations of antibiotics can be seen in Supplementary Table S6.

#### 3.2.3 Combination therapy impact

The stability of most of the antibiotics we studied did not change significantly when combined with other drugs. However, Vancomycin degraded more noticeably in combination (especially with gentamicin), possibly due to incompatibility reactions. Detailed data can be found in Table 2.

**TABLE 2:** Compatibility of drug combination in amino acid dialysate.

Drug name	Single drug (retention rate 4 °C/ 25 °C)	Combination therapy (retention rate 4 °C/ 25 °C)	Conclusion
Ceftazidime	95.7%/ 69.2%	+Cefazolin: 96.5%/ 70.6%	Stability in combination therapy similar to single drug, vancomycin may slightly accelerate degradation
		+Vancomycin: 89.6%/ 75.6%	
Cefazolin	91.3%/ 81.7%	+Ceftazidime: 92.2%/ 84.1%	Gentamicin may have protective effect on cefazolin
		+Gentamicin: 97.2%/ 90.1%	
Vancomycin	72.3%/ 55.5%	+Ceftazidime: 57.0%/ 53.3%	Combination therapy significantly reduces stability, especially when combined with gentamicin
		+Gentamicin: 36.9%/ 28.8%	
		+Heparin sodium: 53.0%/ 49.2%	
Gentamicin	97.0%/ 96.2%	+Vancomycin: 97.0%/ 96.3%	Excellent stability, minimal effect from combination therapy
		+Cefazolin: 96.9%/ 96.6%	
		+Heparin sodium: 95.7%/ 93.9%	
Heparin sodium	99.3%/ 98.6%	+Vancomycin: 99.4%/ 98.6%	Stability unaffected by combination therapy
		+Gentamicin: 99.3%/ 98.6%	
Ampicillin	99.5%/ 84.9%	+Sulbactam (0.5/0.05 mg/mL) 97.8%/ 99.2%/ 82.2%/ 83.3%	Sulbactam has no significant effect on stability, significant degradation at 25 °C

### 3.3 Amino acid changes

In general, the amino acids in the dialysate changed little with time and temperature under different antibiotic environments. Specifically, different amino acids, such as tryptophan, methionine, histidine, histidine hydrochloride are more stable (maximum degradation rate <4%). Threonine and glycine are more susceptible (maximum degradation rate 7.68% and 8.21%). For different drugs, β-lactams seem to have a greater impact on the degradation of amino acids, especially cefazolin (the maximum degradation rates of the ten amino acids appeared in the dialysate mixed with cefazolin, ceftazidime and cefotaxime). Maximum degradation rate of all amino acids are shown in Supplementary Table S7.

### 3.4 Efficacy of gentamicin

Our study only studied the antibacterial efficacy of gentamicin. With prolonged time and increased temperature, the inhibition zone diameters of test samples generally increased, showing an upward trend in potency. After adding amino acids, the antibacterial ability of gentamicin became stronger. The detailed results are shown in Supplementary Table S8.

## 4 Discussion

Our study explored the compatibility of various commonly used antibiotics with amino acid-based peritoneal dialysate. We found that almost all antibiotics were less degraded at 4 °C, and the degradation rate increased with time at all temperature. For specific types of drugs, in amino acid-based peritoneal dialysate, gentamicin, heparin sodium, ciprofloxacin and fluconazole showed excellent stability under different conditions. While meropenem, ceftazidime, cefotaxime, and aztreonam showed significant degradation, especially at high temperature (37 °C). Among the drugs mentioned above, meropenem was the most unstable. In addition, from the perspective of drug categories, compared with other drug types, the stability of β-lactam antibiotics in amino acid dialysis solution seems to be poor. For amino acids, they remained basically stable under different antibiotic environments, with minimal impact on nutritional aspects.

There have been few studies on the compatibility of β-lactam antibiotics in amino acid dialysate. A review of 21 studies found that: some β-lactam agents (including Ampicillin, Cefazolin) were stable for 7–21 days in the refrigerator and 6 h to 14 days at room temperature. However most common β-lactam agents including amoxicillin, aztreonam, cefepime, ceftazidime, imipenem and meropenem yielded less than 6 h of stability in mixed Physioneal solution at body temperature (Jeong et al., 2013). The unstable drugs in amino acid dialysate in our study are basically consistent with the above mentioned drugs. For specific types of drugs, unfortunately, we have not found the corresponding literature as the situation of gentamicin in dialysate. Only the study of Ranganathan et al. showed gentamicin was stable for 14 days at all temperatures (4 °C, 25 °C or 37 °C) in extraneal 7.5% Icodextrin Peritoneal dialysate (Ranganathan et al., 2016). For ciprofloxacin, the study of Kussmann et al. found that whether at 6 °C, 25 °C or 37 °C, ciprofloxacin showed good stability in amino acid dialysate. At its most unstable temperature of 25 °C, its concentration can remain at 87.5% after 14 days (Kussmann et al., 2019). This was consistent with our results. While for meropenem, another study of Kussmann found that the antibacterial efficacy of meropenem decreased significantly in various peritoneal dialysates including amino acid-based peritoneal dialysate (Kussmann et al., 2016). Additionally, previous studies have indicated meropenem yielded less than 6 h of stability in mixed physioneal solution at body temperature (Churchwell, 2023). Our study found that its concentration is also extremely unstable in amino acid-based peritoneal dialysate. In summary, although there is some research evidence (most of these studies included few cases), supporting the use of meropenem in the abdominal cavity (Jeong et al., 2013; de Fijter et al., 2016). It should still be cautious to use meropenem in the treatment of peritonitis in clinical practice. For aztreonam, the study of Tobudic also indicated aztreonam is more stable in extraneal than in amino acid-based peritoneal dialysate. More than a 10% reduction of azetreonam concentration was detected after 7 days in amino acid-based peritoneal dialysate (Tobudic et al., 2020). For cefotaxime, a review of 49 studies reported that prolonged storage at room temperature resulted in instability of cefotaxime in amino acid-based peritoneal dialysate (Bailie and Kane, 1995). This is also consistent with the results we obtained.

The stability of most antibiotics did not change significantly at different concentrations and in combination with other drugs. However, vancomycin shows its uniqueness in these two aspects of our research. Vancomycin at medium concentration (500 mg/mL) showed the poorest stability, but high concentration demonstrated special stability at 4 °C. The study of Deslandes et al. reported that the concentration of vancomycin administered at 30 mg/ L remained stable at 37 °C for 24 h in physioneal dialysate, room temperature (22 °C ± 2 °C) for 12 h and then 37 °C for 12 h in extraneal dialysate (Deslandes et al., 2016). This seems to indicate that vancomycin has good compatibility in dialysate at low concentrations. The study of Ranganathan found that vancomycin (Initial dose 1 g/ L)was stable for 4 days at 37 °C and for 14 days at both 25 °C and 4 °C in extraneal 7.5% Icodextrin peritoneal dialysate (Ranganathan et al., 2016). This is consistent with our results at 4 °C, but is significantly different at 25 °C, which may be due to different types of dialysate, and more studies are needed to confirm. Regarding the initial dose of 500 mg/ L, we did not find relevant studies. However, the above seems to suggest that vancomycin is more suitable for storage in amino acid-based peritoneal dialysate at low initial concentration or high initial concentration at low temperature. For drug combination, our study found that the stability of vancomycin was significantly reduced when it was combined with gentamicin in amino acid-based peritoneal dialysate. There is little research on the interaction between vancomycin and gentamicin concentrations, whether in blood or in peritoneal dialysate. The study of Deslandes et al. indicated that the gentamicin and vancomycin combination was stable for 4 days at 37 °C and for 14 days at 25 °C and 4 °C in extraneal 7.5% Icodextrin peritoneal dialysate. Raverdy et al. mixed vancomycin with gentamicin directly and measured the concentration of the two at 37 °C after 1 h, and also found no incompatibility between the two (Raverdy et al., 2013). Whether it is because the amino acid-based peritoneal dialysate environment in our study has an impact on the results is unknown. Previous studies have also focused on the antibacterial efficacy and adverse reactions of the combination of the two. Vancomycin and gentamicin can be used for the combined treatment of severe infections due to their different antibacterial mechanisms. *In vitro* and clinical studies have confirmed that the two can have a certain synergistic effect in the treatment of methicillin-resistant *S. aureus* (MRSA) and enterococci (Yu et al., 2020; Houlihan et al., 2000). However, some studies have reported that the combination of the two aggravates nephrotoxicity (Goetz and Sayers, 1993). Although the combination of the two is relatively rare in the treatment of peritonitis, it is still hoped that more studies will be conducted in the future to explore the situation of the two in the treatment of peritoneal dialysis patients.

We also found that the antibacterial efficacy of gentamicin in amino acid-based peritoneal dialysate was enhanced. Several studies have demonstrated that amino acids combined with aminoglycoside antibiotics can synergistically reverse bacterial resistance (Jiang et al., 2020; Yong et al., 2021). Guo et al. carried out an *in vitro* and animal experiment using a mixture of amino acids and gentamicin. They found threonine and glycine both had a synergistic bactericidal effect with gentamicin. In terms of mechanism, first, they believe that the TCA cycle pathway of the two groups with these two proteins is more active, and the increase of metabolic flux promotes bacterial metabolism, thus reversing the drug resistance of bacteria. Second, they believe that the addition of these two proteins increases the production of glutamic acid, which has also been reported to reduce bacterial resistance. Both glycine and threonine are contained in the amino acid dialysate, which seems to explain our findings. Unfortunately, we only investigated the antibacterial effect of gentamicin in amino acid dialysate at present, and the change of antibacterial efficacy of other antibiotics is unknown. It is hoped that there will be more related research in the future to provide more basis for clinical medication.

Our research also has some shortcomings. First of all, our study is mainly a screening-level physicochemical stability study, we did not pre-define formal validation protocols (e.g., accuracy, precision, LOD/LOQ) or acceptance criteria (e.g., ±10% for recovery). Instead, we relied on: calibration curves using certified reference standards (r^2^ > 0.999),minternal standard (phenylalanine) for antibiotic assays, and external standard method for amino acids, following standard laboratory practices. We acknowledge that this is a limitation. And there also may be some bias in the actual application to patients, which still need real clinical trials to confirm.

Secondly, we only studied the antibacterial efficacy of gentamicin. We have not studied other antibiotics. In the future, more research is needed to explore the antibacterial efficacy of various antibiotics in amino acid-based peritoneal dialysate, so as to provide more evidence for clinical practice.

In summary, our study demonstrated that most commonly used antibiotics, including gentamicin, ciprofloxacin and fluconazole, are highly stable in amino acid-based peritoneal dialysate and are suitable for intraperitoneal administration. In contrast, β-lactam antibiotics (especially meropenem, ceftazidime and cefotaxime) and medium-to-high concentrations of vancomycin show significant degradation, particularly at room temperature and body temperature, and therefore should be administered promptly after mixing. And we also recommend that all antibiotics should be used as soon as possible or stored at low temperature after mixing with amino acid peritoneal dialysis fluid. Furthermore, amino acids in the dialysate remain largely stable in the presence of these antibiotics, preserving their nutritional benefit. Finally, gentamicin exhibits not only excellent stability but also enhanced antibacterial activity in amino acid dialysate, suggesting a potential advantage for this specific combination. However, vancomycin stability is markedly reduced when combined with gentamicin. Therefore, if a combination of vancomycin and gentamicin is required, the mixture should be administered without delay to avoid underdosing. These findings provide a practical reference for the safe and effective use of intraperitoneal antibiotics in patients receiving amino acid-based peritoneal dialysis.

## Data Availability

The datasets presented in this study can be found in online repositories. The names of the repository/repositories and accession number(s) can be found below: Mendeley Data, doi: 10.17632/dtt2vbmy6j.1 (https://data.mendeley.com/datasets/dtt2vbmy6j/1).

## Appendix

$$\text{Degradation Rate}:\text{Degradation rate }\left(\%\right)=\left(1-0\;h\text{ concentration}/\text{measured concentration}\right)\times 100\%$$

Definitions of grading.

**Table:**

Stability Level	Degradation Rate (14 days 25 °C)	Temperature Sensitivity
Highly stable	≤ 2%	Temperature insensitive
Moderately stable	2%–5%	More stable at low temp
Low stability	10%–19%	Heat sensitive
Unstable	> 19%	Heat sensitive

Temperature Sensitivity	Definition
Heat sensitive	Drugs degrade significantly faster at room temperature (25 °C) and high temperature (37 °C), while being relatively stable at low temperature (4 °C).At 25 °C or 37 °C, degradation rate >10% within 14 days; when stored at 4 °C, degradation rate decreases significantly (30%–50% less than other temperatures)
More stable at low temperature	Drugs show little difference between low temperature and short-term (within 24 h) room temperature (25 °C), but degrade significantly faster at high temperature (37 °C). Degradation rate ≤5% at 4 °C, increases to 5%–10% at 25 °C
Temperature insensitive	Drugs show minimal difference in degradation rate within 4 °C–37 °C range, with temperature changes having extremely small effect on stability. Especially, difference in 14-day degradation rate at different temperatures <2%
