# Supplementary material for: Intraperitoneal administertion: compatibility and stability of common antibiotics in amino acid-based peritoneal dialysate
Source: Front Pharmacol. 2026 Jul 2;17:1808066. doi: 10.3389/fphar.2026.1808066 (PMC13373585; doi:10.3389/fphar.2026.1808066)
Supplement: Supplementary file 1 [file DataSheet1.pdf]

Table S1 High-Performance Liquid Chromatography (HPLC) parameters for antibiotic and amino acid (tryptophan/tyrosine) analysis.

No.	Drug Name	Wavelength	Injection Volume	Gradient
1	Tryptophan	270nm	10μl	
2	Tyrosine	270nm	10μl	
3	Aztreonam	270nm	10μl	
4	Meropenem	220nm	10μl	Gradient 1
5	Ceftazidime	254nm	10μl	
6	Cefotaxime	235nm	10μl	
7	Cefoperazone	254nm	10μl	
8	Ceftriaxone	254nm	10μl	
9	Amoxicillin	254nm	100μl	
10	Fluconazole	254nm	100μl	
11	Piperacillin	220nm	10μl	
12	Tazobactam	220nm	10μl	
13	Ampicillin	230nm	10μl	Gradient 2
14	Sulbactam	230nm	10μl	
15	Ciprofloxacin	278nm	10μl	
16	Cefepime	254nm	10μl	
17	Clindamycin	210nm	10μl	
18	Cefazolin	254nm	10μl	

This table details the detection wavelength and injection volume used for each compound. Two distinct gradient elution programs (Gradient 1 and Gradient 2) were applied as defined in Table S2.

Table S2 Mobile phase gradient programs for HPLC

A Gradient 1

Time (min)	Mobile Phase A (%)	Mobile Phase B (%)
0	97	3
5	97	3
20	78	22
25	75	25
30	30	70
35	30	70
40	97	3
45	97	3

B Gradient 2

Time (min)	Mobile Phase A (%)	Mobile Phase B (%)
0	97	3
5	97	3
30	30	70
35	30	70
40	97	3
45	97	3

Gradient 1 and Gradient 2 were used as referenced in Table S1. Mobile phase A: 0.02 mol/L sodium dihydrogen phosphate; Mobile phase B: acetonitrile.

Table S3 Gradient elution program for amino acid analysis using an amino acid analyzer

Time (min)	Buffer A (%)	Buffer B (%)	Regeneration Solution D (%)	Flow Rate (ml/min)
0	100	0	0	0.45
3.5	100	0	0	0.45
11.0	85	15	0	0.45
17.0	80	20	0	0.45
23.0	75	25	0	0.45
33.0	15	85	0	0.45
41.0	15	85	0	0.45
41.1	0	100	0	0.45
48.0	0	100	0	0.45
48.1	0	0	100	0.45
52.0	0	0	100	0.45
52.1	10	0	0	0.45
65.1	100	0	0	0.45
65.2	100	0	0	0.45

Buffer A: citrate buffer, pH 3.45; Buffer B: citrate buffer, pH 10.85; Regeneration Solution D: sodium hydroxide. Flow rate of the buffer pump was 0.45 mL/min.

Table S4 Column temperature program for amino acid analysis

Time (min)	Column Temperature (°C)
0.0	60
21.0	60
25.0	76
50.0	76
55.0	60
65.0	60

The column temperature was varied during the run as detailed above.

Table S5 Appearance, pH, and Osmolarity of Peritoneal Dialysis Solution

Types of drugs	Concentration administered	Maximum pH change	Maximum change in osmolal pressure (mOsmol/kg)	Appearance change (25 ° C for 14 days)
Cefazolin	500mg/L	0.07	5	Light yellow clear liquid
Cefazolin	125mg/L	0.07	6	Light yellow clear liquid
Ceftazidime	500mg/L	0.07	5	Light yellow clear liquid
Ceftazidime	125mg/L	0.06	7	Yellowish clear liquid
Cefazolin + ceftazidime	500mg/L+500mg/L	0.07	6	Light yellow clear liquid
Vancomycin	25mg/L	0.05	5	Colorless clear liquid
Vancomycin	500mg/L	0.05	5	Colorless clear liquid
Vancomycin	1000mg/L	0.06	5	Colorless clear liquid
Vancomycin + ceftazidime	1000mg/L+500mg/L	0.07	6	Colorless clear liquid
Vancomycin and gentamicin	1000mg/L+20mg/L	0.06	6	Colorless clear liquid
Heparin sodium + vancomycin	500IU/L+1000mg/L	0.05	6	Colorless clear liquid
Gentamycin	20mg/L	0.07	8	Colorless clear liquid
Heparin sodium	500IU/L	0.08	4	Colorless clear liquid
Amoxicillin	150mg/L	0.10	5	Colorless clear liquid with a small amount of precipitation
Ampicillin	125mg/L	0.11	6	Colorless clear liquid
Ciprofloxacin	50mg/L	0.06	5	Colorless clear liquid
Clindamycin	300mg/L	0.06	7	Colorless clear liquid with flocculent material visible to the naked eye
Fluconazole	75mg/L	0.10	7	Colorless clear solution containing extremely small precipitates
Fluconazole	100mg/L	0.10	7	Colorless clear solution containing very little precipitate
Cefazolin + gentamicin	500mg/L+20mg/L	0.08	9	Colorless clear liquid
Meropenem	125mg/L	0.04	5	Light yellow clear liquid
Aztreonam	500mg/L	0.08	6	Slightly yellow liquid with a small amount of precipitation, which disappeared after shaking, and a small amount of flocculent
Aztreonam	250mg/L	0.08	6	Slightly yellow liquid, with a small amount

Types of drugs	Concentration administered	Maximum pH change	Maximum change in osmolal pressure (mOsmol/kg)	Appearance change (25 ° C for 14 days)
				of precipitation, the precipitation disappeared after shaking, with a small amount of flocculent
Cefepime	500mg/L	0.06	6	Light yellow clear liquid
Cefepime	125mg/L	0.06	6	Colorless clear liquid
Cefoperazone	500mg/L	0.06	6	Colorless clear liquid
Cefoperazone	125mg/L	0.06	6	Colorless clear liquid
Cefoperazone	62.5 mg/L	0.06	6	Colorless clear liquid
Cefotaxime	500mg/L	0.07	6	Light yellow clear liquid
Cefotaxime	250mg/L	0.07	6	Colorless clear liquid
Ceftriaxone	500mg/L	0.07	6	Light yellow liquid with a small amount of flocculent
Heparin sodium + gentamicin	500IU/L+20mg/L	0.07	7	Colorless clear liquid
Piperacillin + tazobactam	4 g/L + 0.5 g/L	0.11	9	Colorless solution with a white fluffy blob at the bottom
Piperacillin + tazobactam	1 g/L + 0.125 g/L	0.15	9	Colorless solution with a white fluffy blob at the bottom

The table shows the maximum observed changes in pH and osmolarity, as well as any visible changes in appearance, for each tested mixture over 14 days at 4°C, 25°C, and 37°C. Only changes at 25°C for 14 days are shown as they were the most indicative of instability.

Table S6 Degradation Rate of Antibiotics at 4° C, 25° C, 37° C over Different Time Intervals

Drug Name	Initial Concentration	Degradation Rate(%)										
		4h			24h			48h			14 days	
		4° C	25° C	37° C	4° C	25° C	37° C	4° C	25° C	37° C	4° C	25° C
Cefazolin	125mg/L	0.8779	0.4907	0.8680	0.6540	1.6271	5.2463	0.5551	1.6659	10.3675	5.4283	14.9163
Cefazolin	500mg/L	0.5371	0.3255	0.5984	0.0486	1.1823	5.1267	0.1826	1.5479	10.0392	8.6980	18.2597
Ceftazidime	500mg/L	2.2748	1.5048	3.0112	2.8459	4.7340	16.0143	2.4399	5.7962	26.5247	4.2827	30.7662
Ceftazidime	125mg/L	1.6917	1.3169	1.6480	1.9507	3.8363	12.4896	1.6998	6.1453	23.1053	4.1512	28.4471
Vancomycin	25mg/L	0	1.0180	1.5640	0	0	10.7771	7.4372	0	13.7732	43.4224	41.1579
Vancomycin	500mg/L	0	3.7809	5.2104	0	5.9289	7.2472	0	5.4768	9.4225	45.7784	51.5439
Vancomycin	1g/L	1.2938	0	0.9606	2.6928	0	3.2404	6.4053	0	6.3983	27.6578	44.4857
Gentamicin	1000U/ml	0.3024	0.3325	0.2734	0.6105	0.3910	1.4201	1.2486	1.3336	2.6110	3.5700	3.7651
Heparin Sodium	500mg/L	0	0.1624	0.3295	0.1790	0.3246	0.6312	0.5883	0.6272	0.9649	0.7255	1.3525
Amoxicillin	150mg/L	0.5066	2.4315	1.0983	1.2875	4.2705	9.5731	1.4687	7.0802	19.5237	0	-
Ampicillin	125mg/L	0	0.9932	2.8073	0	4.4164	10.2595	0	3.9001	19.7636	0.5363	15.1489
Ciprofloxacin	50mg/L	0	0	0.1727	0.0977	0	0.1893	0	0.7430	1.0714	0	0
Clindamycin	300mg/L	0	0	0	0	0.2097	0	0	0	0	0	-
Fluconazole	75mg/L	1.6252	3.3007	2.7103	0	0.0995	1.9329	0	2.9679	3.1641	0	-
Fluconazole	100mg/L	2.7402	3.0309	2.7949	0	0.1718	2.5888	0.1131	3.1795	3.2348	0	-
Meropenem	125mg/L	5.8942	4.4337	9.2652	3.7790	12.9406	25.3598	5.9456	28.6583	41.8844	59.9879	84.9736
Aztreonam	500mg/L	0.6518	0.9217	0.9216	0.5592	1.2097	1.3311	0.5629	1.6787	2.5799	6.8353	23.5585
Aztreonam	250mg/L	0.2331	0.7196	1.0615	0.3769	0.7871	1.7880	0.4879	1.1799	2.9252	6.6227	19.0408
Cefepime	500mg/L	0	0.5902	1.8734	1.5328	1.5886	3.8678	1.1069	4.1292	7.4388	0	11.7154
Cefepime	125mg/L	0.3054	1.4670	1.9090	1.6662	3.1904	4.5362	1.0815	6.1989	8.4463	0	12.3751
Cefoperazone	500mg/L	0	1.8423	2.0576	0.7458	3.6861	6.1869	0	5.5055	9.7340	0.5087	13.7630
Cefoperazone	125mg/L	0	0.9506	1.7577	0	2.2303	4.9545	0	4.8593	9.1223	0	13.0786
Cefoperazone	62.5mg/L	0	0	1.2154	0	2.0916	3.8079	0	3.9864	8.7893	0	12.4305
Cefotaxime	500mg/L	0	1.9028	2.0219	0	5.0877	8.6267	0.4035	10.6807	18.5067	0	29.8284
Cefotaxime	250mg/L	0.3843	2.2728	2.4586	0.5366	5.5795	10.1449	1.0778	9.5656	18.8770	0.5817	28.9625
Ceftriaxone	500mg/L	0	0	0.5572	0	1.4866	7.9273	0	3.9758	14.3108	3.6999	18.0853
Piperacillin	4g/L+0.5g/L	0	0.1934	2.5092	0	3.5513	9.8963	0.2243	4.2846	19.5881	2.3927	12.3995
Piperacillin	1g/L+0.125g/L	0	0.5739	3.1039	0	3.1164	10.5406	0	3.8083	20.4220	2.0883	15.7508

Data are presented as the percentage of the initial concentration that degraded.

Table S7 Maximum degradation rate of amino acids

Amino acids	Maximum degradation rate	Maximum degradation rate occurs under conditions
Tyrosine	5.20%	Amoxicillin 25 ° C for 48h
Tryptophan	3.03%	Ceftazidime 37 ° C for 24h
Glycine	8.21%	Cefazolin 4 ° C for 48h
Threonine	7.68%	Cefazolin 4 ° C for 48h
Serine	6.57%	Cefazolin 4 ° C for 48h
Proline	5.85%	Cefazolin 25 ° C for 48h
Alanine	6.90%	Cefazolin 4 ° C for 48h
Valine	5.50%	Cefazolin 4 ° C for 48h
Methionine	2.91%	Cefotaxime 25 ° C for 24h
Isoleucine	6.41%	Cefazolin 4 ° C for 48h
Leucine	6.24%	Cefazolin 4 ° C for 48h
Phenylalanine	6.08%	Cefazolin 4 ° C for 48h
Histidine	3.75%	Ceftazidime 25 ° C for 14 days
Arginine	5.04%	Cefazolin 4 ° C for 48h
Lysine hydrochloride	4.01%	Cefoperazone 25 ° C for 48h
Histidine hydrochloride	2.79%	Ceftriaxone 25 ° C for 24h

This table shows the highest degradation rate observed for each amino acid across all tested conditions (temperatures, time points, and antibiotic combinations) and the specific condition under which this maximum occurred.

Table S8 Antibacterial efficacy of gentamicin before and after mixing with peritoneal dialysis fluid

Temperature - Time	Stage	Inhibition zone diameter (mm)		Difference (mm)	Potency (%)
		Standard	Actual		
4°C-0h	Before mixing	19.89 ± 0.16	19.88 ± 0.14	-0.01 ± 0.02	998.89 ± 1.77
	After mixing	19.99 ± 0.04	20.46 ± 0.09	0.47 ± 0.08	1057.96 ± 13.99
4°C-4h	Before mixing	20.06 ± 0.09	20.03 ± 0.09	-0.04 ± 0.05	995.97 ± 4.49
	After mixing	20.57 ± 0.41	21.15 ± 0.34	0.58 ± 0.15	1067.56 ± 18.48
4°C-24h	Before mixing	19.90 ± 0.03	19.83 ± 0.04	-0.06 ± 0.04	992.89 ± 4.49
	After mixing	20.06 ± 0.39	21.40 ± 0.41	1.34 ± 0.23	1165.92 ± 32.11
4°C-48h	Before mixing	19.89 ± 0.09	19.77 ± 0.09	-0.12 ± 0.03	986.51 ± 3.76
	After mixing	20.37 ± 0.19	21.84 ± 0.33	1.47 ± 0.16	1174.13 ± 18.34
4°C-7day	Before mixing	19.27 ± 0.17	18.99 ± 0.23	-0.27 ± 0.06	966.85 ± 8.19
	After mixing	20.19 ± 0.27	22.06 ± 0.24	1.87 ± 0.12	1226.37 ± 18.75
4°C-14day	Before mixing	19.72 ± 0.05	19.40 ± 0.06	-0.32 ± 0.04	963.32 ± 4.02
	After mixing	19.69 ± 0.19	21.63 ± 0.26	1.94 ± 0.07	1250.70 ± 3.11
25°C-0h	Before mixing	19.50 ± 0.06	19.50 ± 0.04	0.00 ± 0.03	999.62 ± 3.60
	After mixing	20.33 ± 0.35	20.88 ± 0.32	0.54 ± 0.06	1064.93 ± 8.80
25°C-4h	Before mixing	19.53 ± 0.16	19.49 ± 0.18	-0.04 ± 0.03	995.67 ± 3.28
	After mixing	20.32 ± 0.27	20.96 ± 0.33	0.64 ± 0.12	1076.35 ± 14.76
25°C-24h	Before mixing	19.81 ± 0.04	19.77 ± 0.05	-0.04 ± 0.02	995.08 ± 1.73
	After mixing	20.40 ± 0.33	21.91 ± 0.46	1.51 ± 0.17	1177.80 ± 14.50
25°C-48h	Before mixing	19.63 ± 0.13	19.51 ± 0.17	-0.12 ± 0.05	985.67 ± 5.59
	After mixing	20.42 ± 0.27	22.06 ± 0.32	1.64 ± 0.07	1193.39 ± 6.08
37°C-0h	Before mixing	19.58 ± 0.16	19.56 ± 0.17	-0.01 ± 0.02	998.44 ± 1.69
	After mixing	19.66 ± 0.18	20.27 ± 0.17	0.60 ± 0.06	1078.17 ± 8.07
37°C-4h	Before mixing	19.19 ± 0.21	19.16 ± 0.25	-0.03 ± 0.06	996.26 ± 4.44
	After mixing	20.67 ± 0.64	21.66 ± 0.97	0.99 ± 0.63	1117.75 ± 81.81
37°C-24h	Before mixing	20.65 ± 0.04	20.50 ± 0.08	-0.15 ± 0.05	984.80 ± 4.69
	After mixing	20.19 ± 0.28	21.75 ± 0.51	1.56 ± 0.28	1188.05 ± 27.75
37°C-48h	Before mixing	19.65 ± 0.30	19.41 ± 0.33	-0.23 ± 0.04	972.90 ± 5.37
	After mixing	20.64 ± 0.08	22.36 ± 0.25	1.72 ± 0.18	1197.05 ± 19.29

Note: Data are presented as Mean ± SD (n=3).

The potency of gentamicin was assessed using a two-dose microbiological assay against Staphylococcus aureus before and after mixing with the amino acid dialysate. Data are presented as the measured inhibition zone diameters, the difference from standard, and the calculated relative potency (mean of triplicates). The standard curve equations for the assay are provided below.

Staphylococcus aureus (before mixing)				
Standard curve				
5	10	20	40	80
15.79	17.86	19.87	22.08	24.01

Standard curve: **y=6.8631log (x) +10.9929**

Staphylococcus aureus (after mixing)				
Standard curve				
5	10	20	40	80
16.39	18.49	20.15	22.21	24.19

Standard curve: **y=6.41797log(x) +11.9360**

Difference: Actual diameter of inhibition zone -diameter of inhibition zone of standard
potency: y (actual) /y (standard) *1000
